# Percutaneous Biliary Stent Placement in Palliation of Malignant Bile Duct Obstruction

**DOI:** 10.4021/gr2009.10.1315

**Published:** 2009-09-20

**Authors:** Feng Liu, Chun Qing Zhang, Guang Chuan Wang, Fu Li Liu, Hong Wei Xu, Lin Xu, Kai Feng

**Affiliations:** a Department of Gastroenterology, Provincial Hospital Affiliated to Shandong University, Jinan, China 250021

**Keywords:** Stent, Obstructive Jaundice, Percutaneous Transhepatic Biliary Drainage, Biliary Obstruction

## Abstract

**Background:**

To summarize the experiences with the technique of percutaneous biliary stent placement for treatment of malignant biliary obstruction in patients with different types of biliary obstruction.

**Methods:**

Percutaneous biliary stent placement was performed in 126 patients with malignant biliary obstruction. The etiology included 56 cases of cholangiocarcinoma, 28 cases of pancreatic cancer, 12 cases of ampullary carcinoma, 10 cases of primary hepatic carcinoma, 8 cases of gastric cancer metastasis, 6 cases of gallbladder carcinoma, and 6 cases of liver metastasis of colon cancer. The obstructed lesion predominantly involved the common bile duct in 42 patients, common hepatic duct in 39 patients, and hilar bile duct in 45 patients. When the bile duct was punctured successfully under fluoroscopy, the guide wire was explored to across the obstruction segment under the assistant of catheter, then the stent was inserted along the super-slippery guide wire. In patients with hilar hepatic duct lesions involving both left and right hepatic ducts, the both ducts were punctured and bilateral stenting was performed. A 8.5 F internal/external drainage catheter was inserted. The liver function test and ultrasound were performed one week after the procedure to observe the decrease of bilirubin and alleviation of biliary obstruction.

**Results:**

A total of 166 stents were implanted in 126 patients. In the 42 patients with common bile duct obstruction, each patient was implanted one stent. In the 39 patients with common hepatic duct obstruction, each patient was impanted one stent. In the 45 patients with hilur bile duct obstruction, 38 patients were placed 2 stents, one patient was placed with 3 stents, and the rest were placed with one stent. The serum total bilirubin decreased from 309.2 ± 158.3 µmol/L before the procedure to148.5 ± 98.0 µmol/L one week after the procedure (P < 0.001). Alkaline phosphatase and alanine aminotransferase significantly decreased (P < 0.001). Five cases died within 1 month (4%) after the procedure. Complications occurred in 9 cases (7.1%). Six patients underwent combined duodenal self-expandable metal stent placement successfully.

**Conclusions:**

The percutaneous biliary stent placement is a safe and effective palliative therapy for malignant biliary obstruction by improving liver function and 1ife quality.

## Introduction

Malignant obstructive jaundice is a common disease. Most malignant tumors causing bile duct obstruction, such as pancreatic adenocarcinoma, gallbladder carcinoma or cholangiocarcinoma, have a very poor prognosis. At the time of diagnosis, the majority of these tumors are unresectable or the patient can not tolerate surgery, so the palliative treatment is the only option. In 1974, percutaneous transhepatic biliary drainage was first carried out by Molnar et al [1] in the palliative treatment of malignant bile duct obstruction. Since then, the percutaneous transhepatic biliary drainage by drainage tube or stenting has become one of the major treatment methods in patients with unresectable or inoperable malignant biliary obstruction. The clinical application has become more and more popular because of its microtrauma, high success rate, and significant effect. Since June 2003, percutaneous transhepatic biliary stenting was carried out in our hospital, 126 patients suffering from different kinds of biliary obstruction were treated and achieved satisfactory palliative effect. A preliminary summary of the technique and therapeutic effect in treating malignant obstructive jaundice in different parts of the bile duct are presented in this article.

## Patients and Methods

Indications of this procedure include: (1) Obstructive jaundice due to unresectable pancreatic cancer; (2) Primary biliary malignant tumors, including the tumor which has invaded the hilar confluence; (3) Obstructive jaundice caused by advanced liver cancer; (4) Metastatic tumors of hilum, in which bile duct is oppressed by tumor or enlarged lymph nodes; (5) Patient with high surgical risk resulting from a variety of factors, such as old age, general asthenia, poor pulmonary or cardiac function, or the surgery is technically difficult due to the complex anatomy of the surgical region; (6) Patients who can not tolerate ERCP.

Contraindications of this procedure are as the followings: (1) Patients with poor systemic status, such as serious coagulation disorder; (2) Ascites, a relative contraindications; (3) Patients with a wide range of intrahepatic bile duct stricture; (4) Patients with severe stenosis of the portal vein adjacent to the narrow site, the external drainage is not contraindication, but stent implantation is considered to be a contraindication; (5) Permanent stent implantation is considered to be a contraindication in benign stricture.

From June 2003 to October 2008, 126 patients (43 females and 83 males, age ranged from 35 to 85 years, mean age 65.6 years) with inoperable malignant biliary obstruction were treated in our hospital with percutaneous transhepatic biliary placement of metallic stents. All the patients were suffering from main clinical manifestations of various degrees of jaundice. At least one imaging of ultrasound, computed tomography (CT) or magnetic resonance cholangiopancreatography (MRCP) was made before procedure to confirm the extent of obstructive biliary dilatation. Blood clotting time, liver function and blood cell count were carried out, an increased serum total bilirubin (TBIL) of 309.2 ± 158.3 µmol/L (mainly direct bilirubin) was seen. Alkaline phosphatase (AKP) increased to 448.27 ± 257.2 IU/L, and alanine aminotransferase (ALT) was 145.8 ± 106.4U/L. The etiology includes 56 cases of cholangiocarcinoma, 28 cases of pancreatic cancer, 12 cases of ampullary carcinoma, 10 cases of primary hepatic carcinoma, 8 cases of gastric cancer metastasis, 6 cases of gallbladder carcinoma, and 6 cases of liver metastasis of colon cancer. The obstructed lesion predominantly involved common bile duct in 42 patients, common hepatic duct in 39 patients, and the hilar bile duct in 45 patients.

### Instruments

The basic instruments include a 22G Chiba needle, 0.018" micro-guide wire, percutaneous transhepatic biliary drainage (PTBD) trocar, Terumo super-lubricity guide wire, Amplatz super-hard guide wire, 5 F Cobra or multipurpose catheter; 8 - 8.5 F external drainage tube and 8 - 8.5F internal/external drainage tube with multi-side holes for biliary drainage. Special instruments for biliary forming include titanium-nickel self-expandable metallic biliary stents (diameter 8 -10 mm, length 40 – 80 mm, products of Micro-Tech Co, Ltd, and GRIKIN Advanced Materials Co., Ltd ), and balloon catheter (diameter 6 – 8 mm, length of 4 cm). Instruments used for duodenal obstruction included self-expandable metal stent (diameter 18 – 25 mm, length 60 – 80 mm, product of Micro-Tech (Nanjing, China).

### Procedures

**Figure 1:**
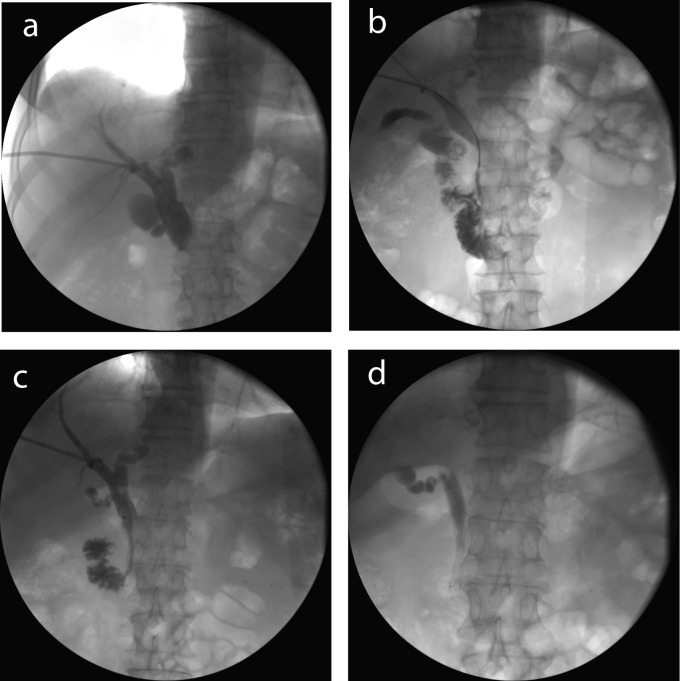
Stent placement in a patient with common bile duct obstruction with pancreatic cancer. (a) Percutanous transhepatic cholangiography showed an obstruction about 2.5 cm in common bile duct; (b) A catheter was advanced through the obstruction under guidance of a guide wire; (c) A 8 mm × 60 mm stent was placed to support the obstruction; (d) Seven days later, the drainage tube was removed, the stent stayed in the bile duct.

The location and extent of obstruction were confirmed by enhanced CT or MRCP preoperatively. The accessing route and puncture point were determined under fluoroscopy. When the bile duct was punctured successfully by a 22G Chiba needle, cholangiography was made to clarify the extent of intrahepatic biliary dilatation (Fig. 1). Then micro-guide wire was put in through the Chiba needle to the hilum or just above the obstruction. The puncture channel was dilated by PTBD trocar along the micro-guide wire to the hilar. Then the inner core of trocar and the micro-guide wire were quitted, and the super-lubricity guide wire through the trocar was fed into the top of obstruction. A 6 F vascular sheath was implanted along the guide wire. The guide wire explored to across the obstruction segment under the assistant of 5F Cobra catheter or multipurpose catheter. If the obstructive segment can be entered, radiography was performed again in order to determine the length of obstruction. The guide wire was put into the duodenum and exchanged to super-slippery guide wire. The stent delivery catheter installed with the stent was advanced to the stenosis carefully along the super-slippery guide wire under fluoroscopy. Then the stent was released carefully, its expansion and patency was observed by a radiography. If the stent implantation was difficult due to severe stenosis, the striction was dilated by balloon catheter before the stent was implanted. In patients with obstruction at the level of the bifurcation, with the left and right hepatic duct isolated, both the left and right ducts were punctured, and bilateral stent implantation was performed simultaneously. In some patients, the guide wire could not pass through the obstruction, or the biliary tract infection existed, an external drainage tube was inserted along the guide wire. After 3 - 5 days drainage, exploration was performed again, and the stent was implanted. Finally, a 8.5 F internal/external drainage tube was inserted along the guide wire, and fixed. Postoperative antibiotics were used routinely. About 3 - 7 days later, the internal/external drainage was removed if the stent’s expansion and patency were well on cholangiography.

In patients suffering from biliary obstruction combining with duodenal obstruction, combined duodenal self-expandable metal stent placement was performed. The extent of duodenal obstruction was realized by endoscopy and X-ray. After the implantation of duodenal stent, percutaneous transhepatic puncture was performed. The guide wire went across the stenosis successfully assisted by catheter into the duodenum through the mesh of the duodenal stent. The narrow section and the mesh of the duodenal stent were expanded by the balloon catheter, and the biliary stent was placed.

### Follow-up

If the patient had no sign of biliary tract infection, such as fever, and the color of the drainage fluid was normal, a cholangiography was done from the internal/external drainage tube 3-7 days after stent placement to make sure the patency and position of stents. If the stent expanded well, the drainage tube was removed. Examination of liver function and ultrasound was done one week after the operation to investigate the decrease of bilirubin and recovery of bile duct obstruction, and then the patients were followed-up regularly every 1-3 months.

## Results

The stent placements in 126 cases were all successful, a total of 166 stents were implanted. In the 42 cases with common bile duct obstruction, one stent were implanted in each patient, among these cases, 29 were placed through the papilla of Vater, 13 were placed above the papilla; in 23 cases, stents were implanted at the first attempt, while the other 19 cases were implanted after 3-5 days' drainage. In the 39 cases with obstruction of the common hepatic duct, one stent was implanted in each patient. In the 45 cases with hilur bile duct obstruction, there was one case placed with 3 stents, 38 cases placed with 2 stents, and the other placed with one stent.

Systemic pruritus was significantly improved after the procedure in all the patients. When cholangiography was done 3 - 7 days later, no stents migration was found. The contrast agent could smoothly enter the intestinal tract, except in one patient the left hepatic duct stent expanded poorly, after balloon dilatation, the position and expansion of the stent was corrected, the contrast agent entered the intestinal tract smoothly.

The blood serum biochemical indicators before operation and 1 week after operation are shown in Table 1. Postoperative serum total bilirubin was 148.5 ± 98.0 µmol/L, it decreased significantly compared with that of preoperative (P < 0.001). The bilirubin decreased significantly in 98 case (decrease by > 50%), partially in 25 cases (decrease by 25% - 50%), slightly in three cases (decrease by < 25%). AKP and ALT also decreased significantly (P < 0.001). Five cases died within 1 month (4.0%) after operation, one case of those presented as a sudden irregular breath and immeasurable blood pressure, and died during the operation, resulting in a procedure-related mortality rate of 0.8%. The reason for death was considered to be a delayed iodine allergy-induced anaphylactic shock. One elder patient (83 years old) died of cardiac arrest 11 days after stent implantation. Two other cases died of tumor metastasis and multiple organ failure 15 days and 20 days postoperatively. One case suffering from liver cancer developed to hepatic encephalopathy postoperative and died of liver failure.

**Table 1:** The bilirubin level and liver function before and 7 days after operation

	Preoperative (n = 126)	Postoperative (n = 126)	P value
TBIL (µmol/ L)	309.2 ± 158.3	148.5 ± 98.0	< 0.001
DBIL (µmol/L)	149 ± 74.9	70.3 ± 44.0	< 0.001
AKP (IU/L)	488.3 ± 257.1	219.17 ± 119.8	< 0.001
ALT (U/L)	145.8 ± 106.4	65.4 ± 41.9	< 0.001

AKP, Alkaline phosphatase; ALT, alanine aminotransferase; TBIL, total bilirubin; DBIL, direct bilirubin

Complications occurred in 9 cases (7.1%). In one patient with a severe abdominal pain, angiography from the external drainage tube showed that the left hepatic duct stent expanded poorly, and contrast agent could not enter the intestinal tract smoothly. After a balloon dilatation, the stent expanded well and the abdominal pain relieved. Four patients developed hemobilia which were controlled by changing the drainage tube with a new one from a new puncture channel, while the previous puncture channel was clogged with steel coli and gelfoam pellets. Liver abscess occurred in one patient who complicated with diabetes, the abscess was cured by repeat puncture and antibiotics injection to the abscess in 74 days. One case developed pleural infection, angiography indicated a bilia-pleural fistula, which was cured by thoracic drainage. Two patients developed biliary peritonitis. Eight patients developed stents reocclusion (6.3%), all of them were treated by plastic stenting by ERCP, drainage tube inserting or stent placement successfully, the time to reocclusion was 54, 82, 98, 107, 124, 285, 285, 360 days, respectively.

In the six patients undergoing combined duodenal self-expandable metal stent placement, all stents were placed successfully, both the symptom of duodenal obstruction and biliary obstruction relieved significantly. All the patients were able to tolerate a solid diet. No immediate stent-related complications and stent migration were noted.

## Discussion

The most effective treatment for malignant obstructive jaundice is tumor resection surgically and biliary-enteric anastomosis. However, because this disease is occult, the chance of surgical resection has usually been lost when diagnosed. In addition, the operative mortality is high at 2% - 5%. Percutaneous transhepatic biliary drainage and self-expandable metal stent placement is a commonly used method for palliative treatment for obstructive jaundice which can significantly improve the quality of life, and extend the survival time.

It is important to choose an appropriate approach as it can improve the success rate, reduce the trauma to the patient, and reduce the incidence of complications. The intrahepatic access route should avoid liver tumor (especially malignant ones). The right approach is chosen generally, because of the thick bile duct and the large volume of the right lobar. When the stricture is located above the convergence, resulting in the isolation of right anterior, right posterior branch or the right lobe atrophy, or the portal vein of the right side has been invaded, a left approach should be chosen to drain more liver parenchyma. In patients with ascites, a left-sided drainage may be preferable, because it might avoid ascites leaking around the catheter which causes skin irritation [2].

In patients with malignant obstructions of common bilary duct, which are usually caused by pancreatic carcinoma, distal cholangiocarcinoma, or ampullary tumours, only one access route with only one stent (most commonly used stent with diameter of 8 - 10 mm) is required to obtain complete drainage of the biliary system. A right-sided or a left-sided sub-xyphoid approach can be chosen (usually right-sided). It was reported that, initial patent rates of patient with a stenosis near the Ampulla of Vater could be improved by placing stent across the papilla [3], Hatzidakis et al suggested that in patients with stenosis more than 2 cm above the ampullary, the incidence of cholangitis and operation-related mortality could be reduced by placing stent across the papilla, in the patient had a poor general conditions, or had a life expectancy no more than three months [4]. Therefore, the stent should be placed across the papilla with its distal end in the duodenal in patient with a stenosis near the ampulla. In patient with stenosis of superior and middle segment of common bile duct more than 2 cm above the ampulla, stent should be placed suprapapillary. However, transpapillary methods should be chosen in patients with poor general condition.

Hepatic duct obstructions are usually caused by cholangiocarcinoma, primary hepatic carcinoma, or metastatic liver cancer. Only one access route are required. And the stent are generally located in hepatic duct, with its upper and lower more than 1 mm end over the stenosis, respectively. For high obstruction of common hepatic duct and the hilum, the stent is placed above the papilla to leave the sphincter function intact and to minimize the risk of reflux of intestinal contents into the biliary tree. If the occlusion extends low in the common bile duct, stenting to the duodenum is appropriate [2, 3].

Hilar biliary obstruction is not only difficult to deal with in surgery, but also a technical difficulty in interventional drainage. It is most often caused by hilar cholangiocarcinoma (Klatskin’s tumour), gallbladder cancer growing into the liver and/or hepatoduodenal ligament, advanced gastric cancer, lymphadenopathy in the hepatoduodenal ligament or liver metastases compressing hilar structures. For obstruction at the level of the bifurcation with the left and right hepatic duct still connected, draining only one lobe with metal stent is usually sufficient, one stent is placed with its distal end in common heptic duct and proximal in the drainged bile duct. Although Inal et al reported no significant difference in clinical response to treatment or stent patency rate with unilobar versus bilobar drainage, even in Bismuth type II and III hilar obstructions [5], most authors suggest that survival is better with drainage of both sides of the liver [6]. So bilobar drainage with metal stent is preferred, in patients with obstruction at the level of the bifurcation, with the left and right hepatic duct isolated. In most cases, the contralateral side may be punctured and two stents is implanted from the ipsilateral and the contralateral side into the common bile duct, placed in a “Y” configuration side-by-side. One advantage of this more anatomic “Y” placement is that the stents are both approachable endoscopically should stent occlusion occur. In addition, the patency rate of the “Y” configuration might be improved. If the obstruction does not extend beyond the secondary confluence, and the guide wire can get across the stricture to the contralateral bile duct, stents may be placed from the ipsilateral side to the contralateral side and from the ipsilateral side into the common bile duct or duodenum in a “T” configuration. For cases with multiple obstruction of intrahepatic bile duct, multi-drainage from multi-approach should be preferred. All the bile duct should be drained as completely as possible in patient combined with biliary tract infections. For some cases difficult to achieve completely drainage, the bile duct allowing for drainage of more functional liver should be drained.

In patients suffering from biliary obstruction combining with duodenal obstruction, surgical biliary-enteric anastomosis and gastrojejunostomy technique were usually chosen for palliation. Although the effect of palliative care is better, this "double bypass" operation is associated with a higher rate of morbidity [7], with mortality and morbidity rates ranging from 2% to 5%, and 17% to 37%, respectively [8-11]. Kaw et al described their experience with 18 patients undergoing simultaneous biliary and duodenal self-expandable metal stent placement. Combined metal stenting was successful in 17/18 patients. Of the 17 patients with successful duodenal stenting, 16 received a good clinical outcome, with relief of obstructive symptoms. No immediate stent-related complications and no stent related deaths were noted. Two patients had recurrent biliary obstruction and two other patients had recurrent duodenal obstruction, all of these patients got successful stent reimplantation [12]. In our study, six patients complicated with symptoms of duodenal obstruction, were performed with combined metal stenting. All of the six patients were clinically success, without any stent-related complications and stent-related deaths. Both the symptoms of duodenal obstruction and biliary obstruction relieved significantly. All of the patients were able to tolerate a solid diet, all of the patients were followed up to death due to multiple organ failure, no re-occlusion of duodenal occur. Combined metal stenting of duodenum and bile duct provide a safe and less invasive alternative to surgical palliation with an acceptable clinical outcome. It can expand the indications of percutaneous transhepatic biliary drainage [3]. Though prospective studies comparing combined biliary and duodenal stenting versus surgery are lacking, for patients with poor general condition and are not good candidate for surgical treatment, it provides a good choice.
